# Fourteen-year trends in prescribing patterns for patients with bipolar mania discharged from a public psychiatric hospital in Taiwan

**DOI:** 10.1097/MD.0000000000037270

**Published:** 2024-03-01

**Authors:** Huei-Ping Chiu, YuJu Shih, Ching-Hua Lin

**Affiliations:** a Kaohsiung Municipal Kai-Syuan Psychiatric Hospital, Kaohsiung, Taiwan; b Department of Psychiatry, School of Medicine, College of Medicine, Kaohsiung Medical University, Kaohsiung, Taiwan; c Department of Post-Baccalaureate Medicine, College of Medicine, National Sun Yat-sen University, Kaohsiung, Taiwan.

**Keywords:** bipolar mania, complex polypharmacy, lithium, second-generation antipsychotics, simple polypharmacy

## Abstract

Bipolar disorder is a complex mental illness. Pharmacological therapy, including antipsychotics and mood stabilizers, is the primary treatment approach for manic episode. The study aimed to analyze prescribing patterns over a 14-year period for patients with bipolar mania discharged from a psychiatric hospital in Taiwan. Patients with bipolar mania discharged from the study hospital between 2006 and 2019 (n = 2956) were included in the analysis. Prescribed drugs for the treatment of manic episode, included mood stabilizers (i.e., lithium, valproate, carbamazepine) and any antipsychotics (i.e., second- and first-generation antipsychotics; SGAs & FGAs). Monotherapy, simple polypharmacy, and complex polypharmacy were also examined. Simple polypharmacy was defined as being prescribed 2 different bipolar drugs (lithium, valproate, carbamazepine, and any antipsychotics), while complex polypharmacy at least 3 bipolar drugs. Temporal trends of each prescribing pattern were analyzed using the Cochran-Armitage Trend test. The prescription rates of valproate, SGAs, and complex polypharmacy significantly increased over time, whereas the prescription rates of any mood stabilizers, FGAs, and simple polypharmacy significantly decreased. Prescription rates of lithium and monotherapy did not significantly change. The study highlights the shifts in prescribing practices for bipolar mania. SGAs were prescribed more while FGAs declined, likely due to SGAs’ favorable properties. Complex polypharmacy increased, reflecting the complexity of treating bipolar disorder. Long-term outcomes of these changes require further research.

## 1. Introduction

Bipolar disorder is characterized by chronic and complex mood dysregulation with recurrent symptoms and episodes of mania, hypomania, depression, and mixed mood. Bipolar disorder results in fluctuations in mood, leading to emotional instability, as well as difficulties in thinking and functioning. Additionally, it heightens the likelihood of experiencing disability and premature mortality.^[1]^ Bipolar disorder affects over 1% of the global population.^[2]^ The lifetime prevalence rates for different types of bipolar disorders are estimated at 0.6% for bipolar I disorder, 0.4% for bipolar II disorder, 1.4% for sub-threshold manifestations of bipolar disorders, and 2.4% for the broader bipolar spectrum.^[3]^ In Taiwan, the prevalence of bipolar disorder is approximately 0.42%.^[4]^

According to Diagnostic and Statistical Manual of Mental Disorders, 4th edition text revision (DSM-IV-TR) or 5th edition (DSM-5),^[5,6]^ bipolar disorder encompasses a spectrum of diagnostic subgroups primarily divided according to the severity of mood elevation experienced during acute episodes. Acute bipolar mania is a psychiatric emergency.^[2]^ Patients in acute manic states commonly exhibit elevated mood, impulsivity, agitation, aggression, engaging in risky behaviors, and may also display psychotic features. These symptoms can be severe, resulting in significant functional impairment, hospitalization, burden, and imposing substantial societal costs.^[1,7]^ Due to the profound impact and swift onset of manic symptoms in numerous patients, the primary objective of treatment is to promptly and effectively manage these symptoms.^[8]^

Pharmacological therapy is the cornerstone treatment for acute mania.^[2]^ The main drugs of anti-manic treatments are mood stabilizers (i.e., lithium, valproate, and carbamazepine), and antipsychotics.^[9]^ Treatment selection in evidence-based medicine requires not only considering controlled and observational data regarding treatment efficacy/effectiveness and safety/tolerability, but also integrating individual patient characteristics.^[10]^ The majority of guidelines for treating bipolar mania recommend starting with either antipsychotics or mood stabilizer as the first option, and augmentations of antipsychotics and mood stabilizer are required to achieve immediate therapeutic effects.^[11–13]^ However, in clinical practice, the use of polypharmacy in acute mania is widespread in both inpatient and outpatient settings.^[14,15]^ The decision between monotherapy and polypharmacy can be influenced by several factors, including the occurrence of breakthrough episodes, previous response to monotherapy, the severity of the illness, clinical requirements, medication safety and tolerability, and the personal preferences of the patient.^[11,13]^ There are several reasons to consider prescribing polypharmacy, including inadequate efficacy of the current treatment, psychiatric comorbidities, a history of severe illness progression, a gradual cross-tapering approach during treatment switching, and potential benefits associated with specific combinations.^[16]^

Significant changes have occurred in the treatment of bipolar mania over the past 20 years, and the pharmacological options for treating bipolar mania have expanded.^[17]^ The aim of the current study was to conduct a detailed examination of the prescribing patterns and any notable shifts that occurred among patients diagnosed with bipolar mania who were discharged from a public psychiatric hospital in Taiwan. The study spanned a period of 14 years, from 2006 to 2019, allowing for an analysis of the changes and trends in medication prescriptions over time. By investigating the patterns of pharmacological treatments administered to these patients during their hospitalization and subsequent discharge, the study sought to provide valuable insights into the evolving landscape of mania treatment in the Taiwanese healthcare system.

## 2. Methods

### 2.1. Ethics

This retrospective cohort study was conducted at Kaohsiung Municipal Kai-Syuan Psychiatric Hospital (KSPH), a mental healthcare facility situated in Kaohsiung city, Taiwan. With a total of 2092 psychiatric beds available in the city, KSPH itself possesses 821 beds and provides a wide range of services, including inpatient, outpatient, emergency, and home visit care. Kaohsiung city, situated in the southern region of Taiwan, is home to approximately 2.72 million people. It encompasses both urban and rural districts. The overall population of Taiwan is estimated to be around 23.4 million.

KSPH was chosen as the study site because it has the largest number of acute care psychiatric beds in Taiwan. This selection aimed to improve the representativeness of the study sample. The study received approval from the institutional review boards of the hospital, and all procedures adhered to the ethical guidelines outlined in the Declaration of Helsinki (2013) and Taiwan national legislation, specifically the Human Subjects Research Act. Since this study relied on a register-based analysis using de-identified electronic patient records, written consent was not necessary. Ethical approval was obtained from Institutional Review Boards of KSPH with the reference code KSPH-2020-08.

### 2.2. Subjects

The inclusion criteria for this study consisted of patients who were discharged from acute care wards to the community with a diagnosis of manic episode of bipolar disorder, as defined by either DSM-IV-TR or diagnostic and statistical manual of mental disorders, 5th edition criteria.^[5,6]^ The study period spanned from January 1, 2006 to December 31, 2019. Experienced board-certified psychiatrists established these diagnoses using a comprehensive approach. This approach involved clinical observations, interviews conducted during hospitalization, review of previous medical records, and information provided by the patients’ primary caregivers.

Several exclusions were implemented for this study. These exclusions included patients who participated in clinical trials during the study period, individuals with incomplete prescription data, patients who were not prescribed lithium, valproate, carbamazepine, or any antipsychotics at the time of discharge, and individuals who were discharged against medical advice. By implementing these exclusion criteria, the study aimed to ensure the robustness and accuracy of the findings.

### 2.3. Drug treatment

In the cohort of hospitalized patients diagnosed with a manic episode of bipolar disorder, this study specifically examined 4 primary drugs and drug classes prescribed at the time of discharge. These included lithium, valproate, carbamazepine, and antipsychotics. Lithium, valproate, and carbamazepine were categorized as mood stabilizers, while antipsychotics were further divided into second-generation antipsychotics (SGAs) and first-generation antipsychotics (FGAs). The SGAs comprised amisulpride, aripiprazole, clozapine, lurasidone, olanzapine, paliperidone, quetiapine, risperidone, ziprasidone, and zotepine. On the other hand, the FGAs included chlorpromazine, clotiapine, flupentixol, haloperidol, and sulpiride,

Monotherapy referred to the practice of administering only one of the following: lithium, valproate, carbamazepine, or antipsychotics. Simple polypharmacy was defined as being prescribed 2 different mood stabilizers (lithium, valproate, or carbamazepine) or a mood stabilizer plus any antipsychotics at discharge. Complex polypharmacy, as defined in prior studies,^[18,19]^ was characterized by the prescription of 3 or more medications from the following options: lithium, valproate, carbamazepine, and any antipsychotics. In accordance with recommendations from previous research,^[20]^ medications such as benzodiazepines and other sedative-hypnotics (e.g., zolpidem and zopiclone), which are not primarily recognized as core treatment modalities for bipolar disorder symptoms, were excluded from the analysis.

### 2.4. Statistical analysis

Descriptive statistics were utilized to offer a comprehensive summary of the dataset. Continuous variables were presented as means and standard deviations, whereas categorical variables were expressed as absolute numbers and percentages. To determine the presence of statistically significant time trends in prescription rates for different drug patterns at discharge over the study period, the Cochran-Armitage trend test was utilized. Data analysis was performed using SPSS version 27.0 (IBM Corp., Armonk, NY), SAS 9.4 software (SAS Institute Inc, Cary, NC), and MedCalc (MedCalc Software, Belgium). Statistical significance was determined at a *P* value of <.05, indicating the threshold for identifying significant findings.

## 3. Results

### 3.1. Clinical characteristics of participants

A total of 5727 patients with bipolar disorder were discharged from the study hospital during the study period, and 2956 were included in the analysis. The demographic and clinical characteristics of the patients are depicted in Table 1. Out of the 2956 patients for analysis, 1429 (48.3%) were male, whereas 1527 (51.7%) were female. The average age of the cohort was 44.3 ± 13.7 years, and the average length of hospital stay was 58.8 ± 147.6 days. Out of the patients included in the study, 2661 (90.0%) were prescribed with any mood stabilizers, while 2722 (92.1%) were prescribed with any antipsychotics. Among the patients prescribed with monotherapy (n = 493, 16.7%), 64 patients (13.0%) received lithium, 120 patients (24.3%) received valproate, 14 patients (2.8%) received carbamazepine, and 295 patients (59.9%) received any antipsychotics. Additionally, 2028 patients (70.4%) were on simple polypharmacy with either a mood stabilizer plus another mood stabilizer (n = 36, 1.7%) or a mood stabilizer plus any antipsychotics (n = 2046, 98.3%). There were 381 patients (12.9%) were prescribed complex polypharmacy at discharge.

**Table 1:** Clinical characteristics of the study population.

	n	%
Sex		
Male	1429	48.3
Female	1527	51.7
Any mood stabilizers	2661	90.0
Lithium	1125	38.1
Valproate	1811	61.3
Carbamazepine	143	4.8
Any antipsychotics	2722	92.1
SGAs*	1948	65.9
FGAs†	1147	38.8
Monotherapy	493	16.7
Lithium	64	13.0
Valproate	120	24.3
Carbamazepine	14	2.8
Any antipsychotics	295	59.9
Simple polypharmacy‡	2082	70.4
A mood stabilizer + another mood stabilizer	36	1.7
A mood stabilizer + any antipsychotics	2046	98.3
Complex polypharmacy§	381	12.9
	Mean	SD
Age (yr)	44.3	13.7
Length of hospital stay (d)	58.8	147.6

* SGAs = second-generation antipsychotics.

† FGAs = first-generation antipsychotics.

‡ Simple polypharmacy was defined as being prescribed 2 different mood stabilizers (lithium, valproate, or carbamazepine) or a mood stabilizer + any antipsychotics at discharge.

§ Complex polypharmacy was defined as being prescribed ≥ 3 bipolar drugs (lithium, valproate, carbamazepine, and any antipsychotic) at discharge.

### 3.2. Temporal trends in different medication

Table 2 presents various medication prescribing patterns from 2006 to 2019.

**Table 2:** Patterns of drug treatment at discharge among patients with bipolar mania cohort, 2006 to 2019.

	2006	2007	2008	2009	2010	2011	2012	2013
Any mood stabilizers, n (%)	132 (86.8%)	156 (95.1%)	182 (92.4%)	177 (88.1%)	194 (92.4%)	172 (91.0%)	199 (90.5%)	197 (93.4%)
Lithium, n (%)	58 (38.2%)	73 (44.5%)	84 (42.6%)	74 (36.8%)	73 (34.8%)	57 (30.2%)	83 (37.7%)	81 (38.4%)
Valproate, n (%)	70 (46.1%)	82 (50.0%)	108 (54.8%)	119 (59.2%)	136 (64.8%)	125 (66.1%)	139 (63.2%)	147 (69.7%)
Carbamazepine, n (%)	12 (7.9%)	12 (7.3%)	11 (5.6%)	6 (3.0%)	10 (4.8%)	6 (3.2%)	12 (5.5%)	9 (4.3%)
Any antipsychotics, n (%)	132 (86.8%)	141 (86.0%)	177 (89.8%)	191 (95.0%)	183 (87.1%)	168 (88.9%)	198 (90.0%)	194 (91.9%)
SGAs*, n (%)	42 (27.6%)	49 (29.9%)	82 (41.6%)	96 (47.8%)	104 (49.5%)	112 (59.3%)	136 (61.8%)	146 (69.2%)
FGAs†, n (%)	95 (62.5%)	101 (61.6%)	116 (58.9%)	120 (59.7%)	101 (48.1%)	80 (42.3%)	85 (38.6%)	71 (33.6%)
Monotherapy, n (%)	37 (24.3%)	30 (18.3%)	33 (16.8%)	4 (16.9%)	37 (17.6%)	37 (19.6%)	42 (19.1%)	29 (13.7%)
Lithium, n (%)	3 (8.1%)	9 (30.0%)	9 (27.3%)	5 (14.7%)	6 (16.2%)	1 (2.7%)	6 (14.3%)	0 (0.0%)
Valproate, n (%)	10 (27.0%)	11 (36.7%)	8 (24.2%)	4 (11.8%)	14 (37.8%)	18 (48.6%)	15 (35.7%)	15 (51.7%)
Carbamazepine, n (%)	4 (10.8%)	2 (6.7%)	1 (3.0%)	1 (2.9%)	1 (2.7%)	1 (2.7%)	0 (0.0%)	0 (0.0%)
Antipsychotics, n (%)	20 (54.1%)	8 (26.7%)	15 (45.5%)	24 (70.6%)	16 (43.2%)	17 (45.9%)	21 (50.0%)	14 (48.3%)
Simple polypharmacy‡, n (%)	110 (72.4%)	124 (75.6%)	145 (73.6%)	145 (72.1%)	154 (73.3%)	137 (72.5%)	144 (65.5%)	145 (68.7%)
1 antipsychotic + 1 mood stabilizer	107 (97.3%)	123 (99.2%)	143 (98.6%)	145 (100%)	148 (96.1%)	136 (99.3%)	143 (99.3%)	143 (98.6%)
2 different mood stabilizers	3 (2.7%)	1 (0.8%)	2 (1.4%)	0 (0.0%)	6 (3.9%)	1 (0.7%)	1 (0.7%)	2 (1.4%)
Complex polypharmacy^§^, n (%)	5 (3.3%)	10 (6.1%)	19 (9.6%)	22 (10.9%)	19 (9.0%)	15 (7.9%)	34 (15.5%)	37 (17.5%)
Patients discharged, n	152	164	197	201	210	189	220	152
	2014	2015	2016	2017	2018	2019	z	*P*
Any mood stabilizers, n (%)	190 (92.7%)	190 (90.0%)	195 (89.9%)	237 (87.8%)	237 (86.8%)	203 (86.0%)	2.518	**.012**
Lithium, n (%)	97 (47.3%)	101 (47.9%)	86 (39.6%)	87 (32.2%)	96 (35.2%)	75 (31.8%)	1.607	.108
Valproate, n (%)	124 (60.5%)	128 (60.7%)	136 (62.7%)	169 (62.6%)	176 (64.5%)	152 (64.4%)	3.935	**<.001**
Carbamazepine, n (%)	11 (5.4%)	6 (2.8%)	8 (3.7%)	14 (5.2%)	17 (6.2%)	9 (3.8%)	1.087	.277
Any antipsychotics, n (%)	190 (92.7%)	203 (96.2%)	208 (95.9%)	254 (94.1%)	263 (96.3%)	220 (93.2%)	5.139	**<.001**
SGAs*, n (%)	155 (75.6%)	178 (84.4%)	186 (85.7%)	225 (83.3%)	237 (86.8%)	200 (84.7%)	21.902	**<.001**
FGAs†, n (%)	74 (36.1%)	64 (30.3%)	52 (24.0%)	65 (24.1%)	67 (24.5%)	56 (23.7%)	15.320	**<.001**
Monotherapy, n (%)	23 (11.2%)	29 (13.7%)	31 (14.3%)	45 (16.7%)	42 (15.4%)	44 (18.6%)	1.872	.061
Lithium, n (%)	4 (17.4%)	4 (13.8%)	5 (16.1%)	4 (8.9%)	2 (4.8%)	6 (13.6%)	1.899	.058
Valproate, n (%)	4 (17.4%)	3 (10.3%)	4 (12.9%)	6 (13.3%)	4 (9.5%)	4 (9.1%)	4.065	**<.001**
Carbamazepine, n (%)	0 (0.0%)	1 (3.4%)	0 (0.0%)	2 (4.4%)	0 (0.0%)	1 (2.3%)	2.219	**.026**
Antipsychotics, n (%)	15 (65.2%)	21 (72.4%)	22 (71.0%)	33 (73.3%)	36 (85.7%)	33 (75.0%)	5.612	**<.001**
Simple polypharmacy‡, n (%)	147 (71.7%)	137 (64.9%)	151 (69.6%)	196 (72.6%)	183 (67.0%)	164 (69.5%)	1.961	**.005**
2 different mood stabilizers	7 (4.8%)	0 (0.0%)	0 (0.0%)	4 (2.0%)	4 (2.2%)	5 (3.0%)	0.731	.465
1 mood stabilizer + any antipsychotics	140 (95.2%)	137 (100%)	151 (100%)	192 (98.0%)	179 (97.8%)	159 (97.0%)		
Complex polypharmacy‡, n (%)	35 (17.1%)	45 (21.3%)	35 (16.1%)	29 (10.7%)	48 (17.6%)	28 (11.9%)	4.755	**<.001**
Patients discharged, n	211	205	211	217	270	273		

Bolded values are statistically significant.

* SGAs = second-generation antipsychotics.

† FGAs = first-generation antipsychotics.

‡ Simple polypharmacy was defined as being prescribed 2 different mood stabilizers (lithium, valproate, or carbamazepine) or a mood stabilizer + any antipsychotics at discharge.

‡ Complex polypharmacy was defined as being prescribed ≥ 3 bipolar drugs (lithium, valproate, carbamazepine, and any antipsychotic) at discharge.

**Figure 1.**
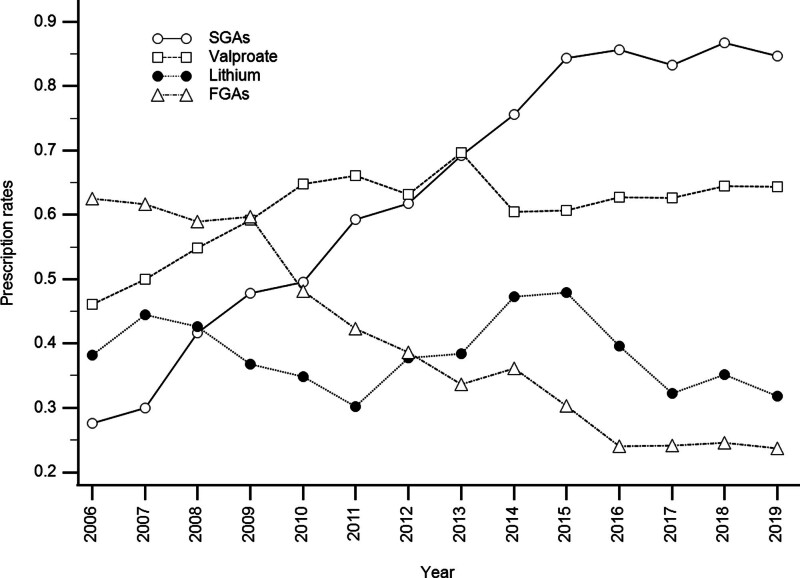
Temporal trends in the prescription rates of lithium, valproate, second-generation antipsychotics (SGAs), and first-generation antipsychotics (FGAs) among patients with bipolar mania at discharge, 2006 to 2019.

The results demonstrated a significant decrease in the prescription rates of any mood stabilizers (*P* < .012), while a significant increase in the prescription rates of any psychotics (*P* < .001) over time. In addition, valproate use significantly increased (*P* < .001), while lithium (*P* = .108) and carbamazepine (*P* = .277) uses did not significantly change. For patients taking any antipsychotics during the study period, SGAs use significantly increased (*P* < .001), while FGAs use significantly decreased (*P* < .001). Figure 1 demonstrates the temporal trends for lithium, valproate, SGAs, and FGAs at discharge from 2006 to 2019.

**Figure 2.**
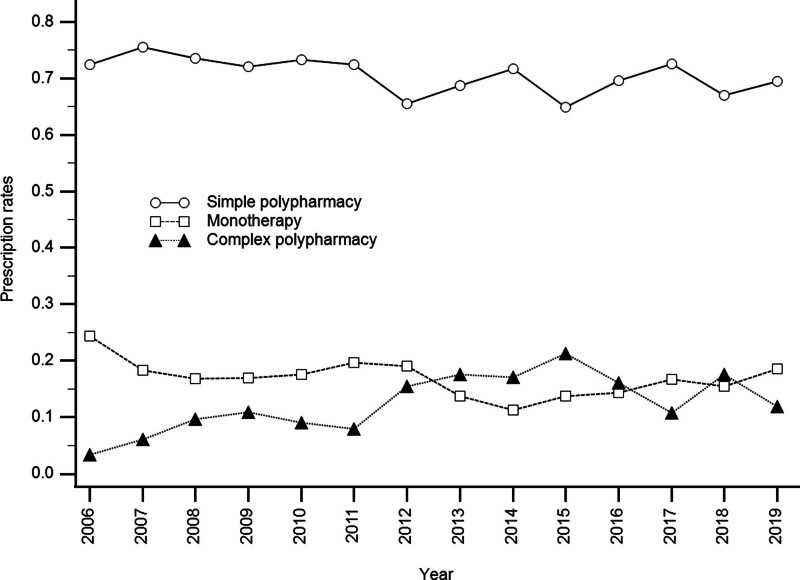
Temporal trends in the prescription rates of monotherapy, simple polypharmacy, and complex polypharmacy among patients with bipolar mania at discharge, 2006 to 2019.

During the study period, simple polypharmacy prescription significantly decreased (*P* = .005), while complex polypharmacy prescription significantly increased (*P* < .001) and monotherapy use did not significantly change (*P* = .061). For patients receiving monotherapy, valproate (*P* < .001) and carbamazepine (*P* = .026) uses significantly decreased (*P* = .005), while antipsychotic use (*P* < .001) significantly increased and lithium use did not significantly change (*P* = .058). For patients receiving simply polypharmacy, both 2 different mood stabilizer and a mood stabilizer plus any antipsychotics uses did not significantly change (*P* = .463). Figure 2 illustrates the temporal trends for monotherapy, simply polypharmacy, and complex polypharmacy at discharge from 2006 to 2019.

## 4. Discussion

The findings of this study revealed significant shifts in the drug management of patients with bipolar mania over a span of 14 years (Table 2). Valproate use and SGA use have been rising over time, whereas FGA use has been decreasing. Most of patients received simple polypharmacy for treatment, especially, a mood stabilizer plus any antipsychotics. The prescribing of complex polypharmacy has increased over time.

### 4.1. Mood stabilizers

The overall prescription rates for any mood stabilizers experienced a slight decline from 86.8% in 2006 to 86.0% in 2019 (*P* = .012). The prescription rates of lithium and carbamazepine did not change during the study period. In contrast, there was a notable increase in the prescription rate of valproate from 46.1% in 2006 to 64.4% in 2019 (*P* < .001) (Table 2).

Lithium and valproate are recommended as first-line treatment options for acute mania.^[13]^ Compared to prescription rate of valproate (61.3%), the lower prescription rates of lithium (38.1%) and carbamazepine (4.8%) could potentially be attributed to its safety and tolerability risks. Lithium exhibits inferior tolerability for side effects such as vomiting, tremor, and thyroid-stimulating hormone (TSH) elevation.^[10]^ One explanation of the low use of carbamazepine is its association with severe skin conditions such as Stevens-Johnson Syndrome or toxic epidermal necrolysis.^[21]^ To ensure patient safety, it is recommended to refrain from prescribing carbamazepine to individuals of Asian descent who possess the human leukocyte antigen (HLA) allele HLA-B*1502, as this biomarker indicates an increased risk of developing a severe rash.^[10]^ In Taiwan, there has been a mandatory implementation of pharmacogenetic testing for specific allelic variants of HLA-B*1502^[22]^ before initiating carbamazepine treatment, especially if this allele has not been detected previously. Therefore, carbamazepine is often considered a second-line treatment option.

The prescription of valproate increased over time. The reasons may be that valproate appears to have broader spectrum of efficacy than lithium, yielding benefit in patient with histories of lithium failure^[23]^ and in patients with lithium-resistant illness subtypes, such as dysphoric mania episodes and multiple prior episodes.^[24]^ Briefly to say, valproate is an important treatment option for acute mania that may have efficacy and/or tolerability advantages over lithium.^[10]^

### 4.2. Antipsychotics

The prescription rates for any psychotics experienced an increase from 86.8% in 2006 to 93.2% in 2019 (*P* < .001). Notably, there was a significant growth in the prescription rate of SGAs from 27.6% in 2006 to 84.7% in 2019. This trend was accompanied by a decline in the use of FGAs from 62.5% to 23.7% (*P* < .001) (Table 2). When considering the decline in the prescription of FGAs, adverse effects emerge as a significant apprehension. Haloperidol, for instance, exhibited poor tolerability and carried a high risk of akathisia and extrapyramidal symptoms.^[25]^ For much of the 1970s and 1980s, FGAs were commonly used (often in combination with lithium) for the treatment of acute mania, but such usage changed as the tolerability limitations of FGAs became more evident and as new treatment options emerged.^[10]^ In the late 1990s and early 2000s, the SGAs overtook FGAs in treatment of acute mania primarily due to tolerability concerns of FGAs and the impressive efficacy evidence base accumulated for SGAs.^[10]^ Rising trend of SGAs use over FGAs, as observed in our study, corresponds to the trends observed in a 7-year analysis of prescription patterns for bipolar mania conducted by Kleimann et al in Europe,^[17]^ which revealed a rise in use of SGAs during the period of 2005 − 2011, from 70% to 79% (*P* < .005), while use of FGAs decreased from 19% to 13% (*P* < .05). Besides the SGAs’ preferable adverse effects, SGAs have been found to be effective for the treatment of bipolar depression, alone or in combination with mood stabilizers and/or antidepressants. They also have efficacy for maintenance treatment in bipolar I disorder.^[13,26]^ Therefore, SGAs have been increasingly available and used more often generally. In short, our study findings align with this prescription shift, highlighting the evolving utilization patterns and preferences in antipsychotic treatment for acute mania.

Based on well-designed clinical trials and comprehensive network meta-analyses, numerous SGAs exhibit favorable antimanic properties and are generally well-tolerated. Almost all antipsychotics are effective in treating mania, with the more potent dopamine D2 receptor antagonists such as risperidone and haloperidol demonstrating slightly higher efficacy.^[25]^ Notably, aripiprazole, olanzapine, quetiapine, and risperidone have demonstrated efficacy in treating acute mania, surpassing the placebo in terms of acceptability.^[25,27]^ The administration of antipsychotic agents or mood stabilizers is widely recognized as the primary approach to pharmacological treatment for acute mania.^[28]^ While antipsychotics have shown effectiveness in improving psychotic symptoms, mood stabilizers like carbamazepine, lithium, and valproate have not demonstrated the same efficacy in this regard. Therefore, individuals presenting with psychotic features should be considered for treatment with antipsychotics.^[25]^ Furthermore, a recent network meta-analysis corroborated the effectiveness of lithium and valproate in managing mania symptoms; however, these mood stabilizers yielded lower efficacy results when compared to the majority of antipsychotics.^[25]^ Furthermore, compared with mood stabilizing medications, SGAs have a faster onset of action, making them a first line treatment for more severe manic symptoms that require rapid treatment.^[29]^ A comprehensive analysis of double-blind randomized controlled trials focusing on bipolar mania conducted a systematic review and network meta-analysis.^[25]^ The findings indicated that among the various treatment options, only SGAs exhibited higher levels of acceptability compared to the placebo. Therefore, clinicians may prefer to use SGAs as first-line treatments for acute mania.

### 4.3. Monotherapy

Ony 16.7% of manic patients received monotherapy for treatment. Monotherapy did not change during the study period (*P* = .061). Classical mood stabilizers, namely lithium, valproate, and carbamazepine, are widely used as monotherapy for acute mania. It has been reported that only a portion of patients with manic episode will respond to monotherapy.^[30,31]^ Valproate monotherapy decreased from 27.0% in 2006 to 9.1% in 2019, whereas antipsychotic monotherapy increased from 54.1% in 2006 to 75.0% in 2019. The rising prescription of antipsychotics as monotherapy over time may reflect higher efficacy of antipsychotics than mood stabilizers in the treatment of bipolar mania.^[25]^

### 4.4. Simple polypharmacy

Although there has been a slight decrease in the prescription of simple polypharmacy (from 72.4% to 69.5%), 70.4% of patients received simple polypharmacy in the treatment of bipolar mania at discharge. In overview of 22 acute mania studies, response rates for 2-drug combination therapy (with olanzapine, risperidone, quetiapine, aripiprazole, or asenapine added to lithium or valproate) exceed those of monotherapy (with lithium or valproate) by approximately 18%.^[10]^ In the clinical practice, for patients with acute mania, if there is no response to monotherapy after 1 to 2 weeks, a combination of different drugs may be considered. That being said, monotherapy is often not sufficiently effective for acute and/or maintenance therapy.^[32]^ Simple polypharmacy is required in situations where a rapid response is necessary, or in patients who are considered high-risk, have previously exhibited partial response to monotherapy, or are experiencing more severe manic episodes.^[13]^ This is the interpretation of the limited use of monotherapy in hospitalized patient with acute mania due to severity of their manic episodes. Simple polypharmacy involving antipsychotics and mood stabilizers was demonstrated to have superior efficacy to antipsychotic monotherapy.^[10,30]^ The evidence suggests that for acute mania a combination of lithium or valproate and an antipsychotic is the most effective approach, with approximately 20% more patients responding to the combination than to monotherapy with any antimanic agent.^[33]^ The combination of an antipsychotic agent and a mood stabilizer, especially for severe mania, appears to be more efficacious than either medication alone.^[16]^ The combination of 2 different mood stabilizers is recommended as a second-line choice.^[13]^ These studies may reflect the findings of our study, which observed a very high percentage of patients (98.3%) receiving a mood stabilizer plus any antipsychotics as simply polypharmacy, while a very low percentage of patients (1.7%) taking 2 different mood stabilizers as combination treatment.

### 4.5. Complex polypharmacy

Prescription rates of complex polypharmacy gradually increased from 3.3% in 2006 to 11.9% in 2019. This trend aligns with a nationwide and population-based study showing that the multiple-drug treatment regimens for bipolar patients have increased over the past decade.^[34]^ There are some explanations for increasing complex polypharmacy. First, this may be due to the complexity of treating bipolar disorder. In fact, the presence of different psychopathological and clinical dimensions as well as potential multiple comorbidities (i.e., medical, psychiatric, and substance use) may limit the effectiveness of monotherapy.^[35]^ Second, patients with chronicity, treatment resistance, and comorbid conditions are often transferred to public mental hospitals for treatment.^[36]^ Third, over the past 3 decades, the US FDA has approved multiple psychotropic drugs for bipolar disorder treatment, expanding the prescribing options for psychiatrists.^[37]^ Fourth, psychiatrists may feel safer adding a new psychotropic agent than switching from or discontinuing an original drug with inadequate treatment response.^[38]^

### 4.6. Strengths & limitations

This study possessed several strengths, such as a large sample size, which contributed to a reduced likelihood of type II error. Additionally, the study encompassed long-term observations in real-world treatment settings, further enhancing its validity and relevance.

Nevertheless, it is crucial to acknowledge and address several limitations of this study. First, as a retrospective study, treatment allocation was not randomized, which introduces the potential for bias and confounding variables. Second, the diagnosis of bipolar disorder relied on clinical judgments rather than standardized diagnostic criteria, which may introduce variability and potential inaccuracies. Third, it is important to note that this cohort specifically consisted of patients who had recently been discharged from a psychiatric hospitalization, which differs from a nationwide and population-based study^[34]^ that includes both inpatients and outpatients for analysis. This distinction in the study population should be taken into account when interpreting and generalizing the findings. It is important to acknowledge that the findings of this study may not be applicable to other settings or patient populations. However, it is generally recommended to continue the use of drugs that have demonstrated efficacy in the treatment of bipolar mania as part of maintenance treatment.^[13]^ Since patients typically received discharge after achieving stabilization, the following medications were those on which the patient had stabilized. Our findings may reflect the prescribing trends for long-term maintenance treatment in such patients. Finally, the definition of complex polypharmacy in bipolar patients has been inconsistent across studies,^[18–20]^ thereby limiting the ability to make comparisons with other research.

## 5. Conclusion

The study findings highlight 2 significant shifts in prescribing practices for patients with bipolar mania. First, there was an increase in the prescription of SGAs, accompanied by a decline in the use of FGAs. This shift may be attributed to the favorable tolerability of SGAs compared to FGAs, which are known to cause more extrapyramidal side effects. However, treatment with SGAs is likely to be associated with prominent metabolic syndrome and weight gain. Second, there was a rise in complex polypharmacy which may reflect the complex nature of treating bipolar disorder. However, complex polypharmacy may also increase the risks of medical nonadherence, adverse side effects, drug interactions, medication error, and cardiometabolic comorbidity.^[18]^ Further research is necessary to assess the long-term outcomes associated with these changes in prescribing practices.

## Author contributions

**Formal analysis:** Huei-Ping Chiu, Ching-Hua Lin.

**Methodology:** Huei-Ping Chiu, YuJu Shih, Ching-Hua Lin.

**Visualization:** Huei-Ping Chiu, Ching-Hua Lin.

**Writing – original draft:** Huei-Ping Chiu, YuJu Shih, Ching-Hua Lin.

**Writing – review & editing:** Huei-Ping Chiu, YuJu Shih, Ching-Hua Lin.
